# An After-Hours Virtual Care Service for Children With Medical Complexity and New Medical Technology: Mixed Methods Feasibility Study

**DOI:** 10.2196/41393

**Published:** 2023-11-08

**Authors:** Katherine Babayan, Krista Keilty, Jessica Esufali, Francisco J Grajales III, Connected Care Live for Family Caregivers Advisory Group

**Affiliations:** 1 Institute of Health Policy, Management and Evaluation University of Toronto Toronto, ON Canada; 2 Connected Care Program Hospital for Sick Children Toronto, ON Canada; 3 Child Health Evaluative Studies SickKids Research Institute Toronto, ON Canada; 4 Lawrence S. Bloomberg Faculty of Nursing University of Toronto Toronto, ON Canada; 5 see Acknowledgments

**Keywords:** children with medical complexity, technology dependence, medical devices, family caregivers, virtual care, home and community care, emergency department visits, enteral feeding tubes, hospital-to-home transition, feasibility, mixed methods

## Abstract

**Background:**

Family caregivers (FCs) of children with medical complexity require specialized support to promote the safe management of new medical technologies (eg, gastrostomy tubes) during hospital-to-home transitions. With limited after-hours services available to families in home and community care, medical device complications that arise often lead to increased FC stress and unplanned emergency department (ED) visits. To improve FC experiences, enable safer patient discharge, and reduce after-hours ED visits, this study explores the feasibility of piloting a 24/7 virtual care service (Connected Care Live) with families to provide real-time support by clinicians expert in the use of pediatric home care technologies.

**Objective:**

This study aims to establish the economic, operational, and technical feasibility of piloting the expansion of an existing nurse-led after-hours virtual care service offered to home and community care providers to FCs of children with newly inserted medical devices after hospital discharge at Toronto’s Hospital for Sick Children (SickKids).

**Methods:**

This exploratory study, conducted from October 2020 to August 2021, used mixed data sources to inform service expansion feasibility. Semistructured interviews were conducted with FCs, nurses, and hospital leadership to assess the risks, benefits, and technical and operational requirements for sustainable and cost-effective future service operations. Time and travel savings were estimated using ED visit data in SickKids’ electronic medical records (Epic) with a chief complaint of “medical device problems,” after-hours medical device inquiries from clinician emails and voicemails, and existing service operational data.

**Results:**

A total of 30 stakeholders were interviewed and voiced the need for the proposed service. Safer and more timely management of medical device complications, improved caregiver and provider experiences, and strengthened partnerships were identified as expected benefits, while service demand, nursing practice, and privacy and security were identified as potential risks. A total of 47 inquiries were recorded over 2 weeks from March 26, 2021, to April 8, 2021, with 51% (24/47) assessed as manageable via service expansion. This study forecasted annual time and travel savings of 558 hours for SickKids and 904 hours and 22,740 km for families. Minimal technical and operational requirements were needed to support service expansion by leveraging an existing platform and clinical staff. Of the 212 ED visits related to “medical device problems” over 6 months from September 1, 2020, to February 28, 2021, enteral feeding tubes accounted for nearly two-thirds (n=137, 64.6%), with 41.6% (57/137) assessed as virtually manageable.

**Conclusions:**

Our findings indicate that it is feasible to pilot the expansion of Connected Care Live to FCs of children with newly inserted enteral feeding tubes. This nurse-led virtual caregiver service is a promising tool to promote safe hospital-to-home transitions, improve FC experiences, and reduce after-hours ED visits.

## Introduction

### Background

Children with medical complexity (CMC) are known to have multiple and severe chronic health problems, functional limitations, and a dependence on medical technologies (eg, central lines and tracheostomies) with increased caregiver needs [1-4]. CMC may be discharged to home and community care settings on 1 or more medical devices that they depend on to support vital functions, such as eating and breathing. Among the varied medical devices used in home and community care, enteral feeding tubes are one of the most common technologies and have high rates of unplanned hospital visits [5-8]. An enteral feeding tube is a medical device placed in the nose, mouth, or a stoma that is used to deliver liquid nutrition, medications, or fluids directly into the gastrointestinal tract, such as a gastrostomy, gastrojejunostomy, nasogastric, nasojejunostomy, or jejunostomy tube (G tube, GJ tube, NG tube, NJ tube, or J tube, respectively) [9,10]. The typical feeding tube complications that these patients present with to the emergency department (ED) are displacement, blockage, leakage, bleeding, and infection [4,5]. Increased stress associated with managing feeding complications and frequent trips to seek medical care are common challenges family caregivers (FCs) face while caring for children with newly inserted enteral tubes [11].

Although CMC make up less than 1% of children in Canada, they account for nearly 60% of total pediatric hospital care costs [1]. When complications related to medical devices arise, the associated costs tend to be even higher for CMC and those who depend on technology devices (ie, CMC with technology dependence [CMC-TD]), due to their increased utilization of health services and visits to the ED [1,3,4]. To enable safe medical device management during hospital-to-home transitions, CMC-TD often require highly skilled, around-the-clock care from FCs and home and community care providers [12]. In preparing for discharge, it is important that FCs receive specialized education to enable their confidence and competency in pediatric complex care to prevent and troubleshoot medical device complications, while reducing unplanned ED visits [12]. One of the FCs noted as follows:

My daughter was diagnosed with a rare genetic illness and spent the first year of her life at the hospital. Six years ago, when she was first discharged from the hospital, we had limited support managing her G tube and oxygen therapy. A home care nurse was sent to visit her, but they did not arrive until three days after discharge, so my only option was to call the in-patient unit to ask for their support. Again, more recently, I had an incident in 2020, where my daughter’s G tube had fallen out. I had inserted her emergency catheter but was not able to pull back gastric fluid to get a pH reading. I tried calling the hospital but realized the clinic team was only available during office hours. Then, I called the physician on-call at the hospital, who told me they could not see the G tube, so it was best I came into the hospital. I ended up taking my daughter to the emergency department for a complication that could have been avoided entirely and virtually managed at home with video calling support.

### About the Program and Service

**Figure 1:**
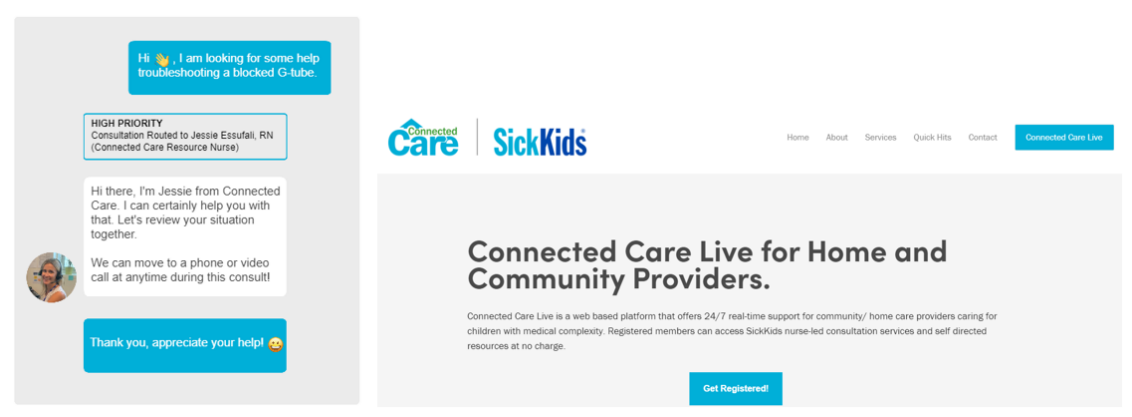
The Connected Care Live virtual care service on mobile and desktop.

At the Hospital for Sick Children (SickKids), Canada’s largest pediatric hospital, over 3000 CMC-TD are discharged each year to home who have an ongoing need for specialized home and community care [13,14]. SickKids partners with home and community care providers to support the unique needs of this population, drive system integration, and promote seamless hospital-to-home transitions through their Connected Care program [14]. Connected Care provides specialized medical device training through an annual program comprising over 2000 in-hospital and virtual family education sessions before discharge, along with more than 1000 education modules tailored for home and community care providers, such as nurses from home care agencies, across Ontario [14]. Home and community care providers can access 24/7 real-time virtual care support through the Connected Care Live service. This support is available via text, phone call, or video call and is provided by an interprofessional team of SickKids clinicians, primarily registered nurses, who are experts in the use of home care technologies (Figure 1) [14]. This web-based service, accessible via smartphones/tablets and PC/laptop, is device agnostic and supports over 1000 providers across the Greater Toronto Area [15]. Connected Care Live consultations are typically used to help users troubleshoot medical device complications; reinforce specialized education and skills; and clarify patient medical orders, care plans, or medications [15]. Currently, this service is exclusively available to regulated care providers in home and community care settings who care for CMC-TD.

After discharge, FCs at SickKids cannot access the Connected Care Live service but are offered specialized support through scheduled virtual visits arranged by Connected Care staff within a week of discharge, self-directed online AboutKidsHealth resources, email and telephone office-hours contact with specialized medical device teams (eg, G-Tube Resource Team), and home and community care provider services [14]. However, virtual visits cannot be initiated by FCs and are not accessible in real time. Besides, many specialized medical device teams are not available after-hours, further limiting support available to FCs during this time. In addition, not all CMC-TD are eligible for home and community care provider services and significant reductions have been seen across home and community care services because of major health human resource shortages, fears of COVID-19 transmission, and greater desire among FCs to increase self-efficacy [16]. With limited access to specialized medical device support during after-hours, a more sustainable strategy is required to support FCs of CMC-TD beyond discharge.

### Study Goal

This study aims to establish the economic, operational, and technical feasibility of piloting the expansion of Connected Care Live to FCs of CMC-TD with newly inserted enteral feeding tubes (ie, G, NG, and GJ tubes) after discharge from SickKids, to ensure the sustainability of a nurse-led after-hours virtual care service for this high-needs population.

## Methods

### Study Design

An exploratory study was conducted from October 2020 to August 2021 using mixed data sources to evaluate the service expansion. A total of 30 semistructured interviews were conducted with key informants including FCs of CMC-TD, nurses, and hospital leadership to assess the risks, benefits, and technical and operational requirements of the service expansion. Economic feasibility was assessed by considering anticipated risks and benefits, which were determined through interviews. We estimated time and travel savings for families, clinicians, and the hospital by comparing the existing state of after-hour services with the anticipated future state following the implementation of Connected Care Live. Similarly, previous studies have used time invested in implementing new technologies as a measure of economic value to establish feasibility [17]. Operational feasibility was assessed by identifying the resources and workflow changes required for service expansion. Technical feasibility was assessed by identifying the technical requirements for delivering the service.

### Semistructured Interviews

A mix of virtual and in-person semistructured interviews was conducted with FCs, nurses, and hospital leadership. Interview participants were self-selected in response to an email and were purposively recruited to ensure the breadth of perspectives was reflected across individuals that had relevant experience with the identified challenges, type of project, or proposed service. The sample size was determined by considering the segmentation of leader viewpoints, diversification of the participants, and saturation achieved through iterative data analysis. A semistructured interview guide informed by principles of child and family-centered care was developed iteratively by the study team [18]. Interview guides were sent out 24 hours in advance of each meeting to promote readiness and respect for participant’s time. At the start of each interview, participants were informed about the purpose and that observational notes would be taken to support the feasibility assessment.

A qualitative content analysis of all interviews was conducted using a conventional approach. This analysis included 3 steps: (1) familiarization with the data through manual reading of text from observational notes taken in response to open-ended questions; (2) identification of key categories related to risks, benefits, and operational and technical requirements described by participants; and (3) selection of quotes and examples [19]. The preliminary analysis of interview content was completed sequentially after data collection by the primary author (KB), which was followed by a secondary analysis performed by 2 coauthors (KK and JE). The identification of categories was driven by quantity and relative importance as expressed by the most common and greatest challenges (eg, gap in after-hours support) identified across interview participants.

### Economic Feasibility

ED visits and after-hours medical device inquiries data were used to estimate the cost of inaction for the time and travel savings analysis. Before data extraction, hospital approval was obtained to extract relevant data from the SickKids’ Epic electronic medical record team. Over a 6-month period from September 1, 2020, to February 28, 2021, data on all ED visits with a chief complaint of “medical device problems” were extracted from the Epic electronic medical record. Variables that were analyzed from these data included type of medical device, reason for visit, and number of hours in ED. The study team also examined and classified each ED visit to identify those that were likely “avoidable” had there been access to a virtual care solution for these FCs. The criteria for visits that could not be managed virtually were developed in consultation with subject matter experts from the Connected Care and G-Tube Resource Team (Textbox 1).

To estimate the time spent managing after-hours emails and voicemails, over a 2-week period from March 26 to April 8, 2021, data on medical device inquiries by FCs to the G-Tube Resource Team were collected. The analyzed variables were mode of contact, date of contact, reason for contact, and disposition. The study team also examined and classified each inquiry, identifying those that could be managed by the Connected Care Resource Nurses. The criteria for Connected Care manageable inquiries were also developed in consultation with subject matter experts from the Connected Care and G-Tube Resource Team (Textbox 2).

**Exclusion criteria for virtually manageable emergency department visits.**

- Obtained imaging (eg, x-rays, ultrasound for tube placement) and tests (eg, nasopharyngeal swabs).
- Required intravenous access or medications not part of the home care plan.
- Obtained blood work (not including blood sugar levels checked for missed feeds).
- Dispositioned to hospital admission.

**Exclusion criteria for medical device inquiries manageable by Connected Care Resources Nurses.**

- Appointment-related questions
- Serious and life-threatening conditions that require immediate medical attention
- Gastrojejunostomy tube dislodgement–specific questions

### Operational Feasibility

ED visits and after-hours inquiries data were used to forecast the volume and timing of Connected Care Live consultation service demands. We measured the service’s operational requirements in hours, using service demand data to estimate the annual nursing full-time equivalents needed for pilot service expansion. This also included the hours required for specialized G tube training for Connected Care Resource Nurses, quality assurance, and administrative tasks before and during the initial pilot. Estimated service demands were compared with existing scheduling and human resource capacity to determine the impact on workflows.

### Technical Feasibility

We assessed technical feasibility by identifying the technical requirements for delivering the service in consultation with internal subject matter experts, including those from Connected Care, privacy, legal, risk, and information security functional departments, during semistructured interviews.

### Ethics Consideration

Ethics approval was not sought for this study, given the minimal risk posed to participants. In its entirety, it is compliant with the Tri-Council Policy Statement (TCPS2 2022 [20]) on research ethics. Participation in data collection was completely voluntary. Before observational notes were taken during interviews, all research participants provided informed consent and were briefed on their right to opt out of the data collection process at any time.

## Results

### Overview of Study Participants

A total of 30 participants were interviewed in this feasibility study. The participants represented a variety of roles that directly and indirectly provide support to CMC-TDs, including FCs, nurses, and hospital leadership (Table 1). All FCs interviewed had experience caring for their children with newly inserted enteral tubes, nurses had anywhere from 5 to 16 years of clinical experience managing medical device complications, and hospital leadership had previous experience supporting the implementation of virtual care services.

**Table 1:** Participants interviewed from SickKids (N=30).

Participant group			Participants, n
**Family caregivers**			
	Parents of children with medical complexity from the SickKids’ Patient and Family Advisory Network	2	
**Nurses**			
	Emergency department	2	
	Connected Care Program	4	
	Gastrostomy Tube Resource Team	3	
**Hospital leadership**			
	Connected Care Program leadership and administrative staff	4	
	Clinical directors and managers	5	
	Functional department leads for privacy, information security, legal, and risk, information management, and technology	6	
	Quality and education leads	2	
	Project managers	2	

### Economic Feasibility

#### Results of the Risk-Benefit Analysis From Interviews With Families, Nurses, and Hospital Leadership

**Figure 2:**
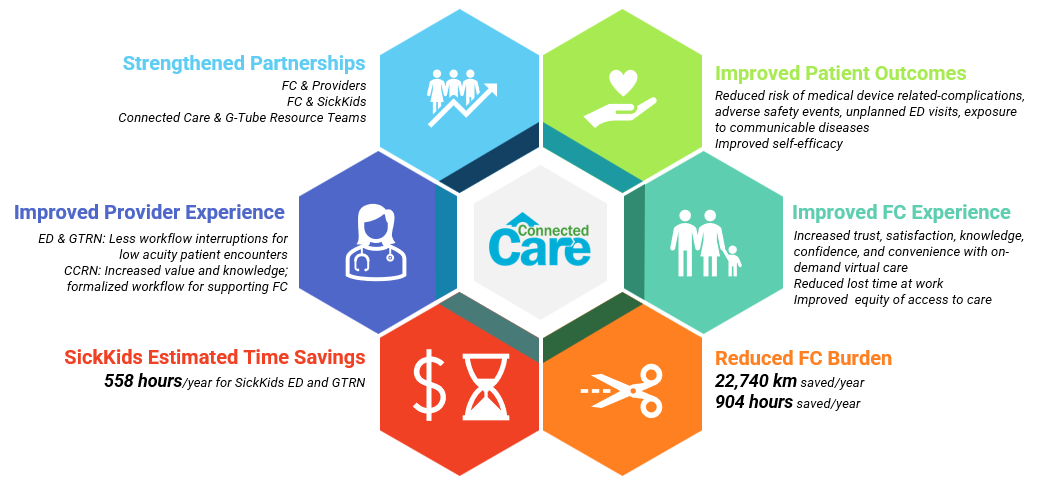
Value emerging from Connected Care Live service expansion to families. CC: Connected Care; CCRN: Connected Care Resource Nurse; ED: emergency department; FC: family caregiver; GTRN: Gastrostomy Tube Resource Nurse.

The risks of service expansion identified by interview participants are shown in Table 2. These included service demand, legal, privacy, technical, nursing practice, and purpose of service. The benefits of service expansion identified by interview participants are shown in Figure 2. These included safer and more timely management of medical device complications, improved caregiver and provider experiences, strengthened partnerships, and significant annual time and travel savings for FCs, SickKids’ ED, and G-Tube Resource Nurses.

**Table 2:** Risks of service expansion to families identified among interview participants.

Risk category	Participants impacted	Risk description
Service demand	Connected Care Resource Nurses Connected Care Leadership	Risk of high-volume and long-duration consults Risk of inadequate staffing and health human resources
Legal	Connected Care Resource Nurses	Risk of providing recommendations that result in potential or actual harm to patients
Privacy	Patients, family caregivers, and Connected Care Resource Nurses	Risk of difficulty verifying end users (authorized family caregivers) to protect personal health information; risk of lost or stolen devices
Technical	Family caregivers and Connected Care Resource Nurses	Risk of having technical issues or service interruptions during consults
Nursing practice	Connected Care Resource Nurses	Risk of errors; concern for gaps in knowledge when supporting family consults
Purpose of service	Patients Connected Care Resource Nurse	Risk of harm for delayed emergency department visits if the family caregiver misunderstands the purpose of the service Risk of increased time and resources spent redirecting family caregivers

#### Emerging Themes

##### Addresses Patient Safety and Gap in the After-Hours Support to Family Caregivers

Many interview participants emphasized the limited follow-up and lack of real-time support available to FCs beyond discharge to enable safe after-hours management of their child’s medical device. Participants highlighted the value that the Connected Care Live service would bring to support families after-hours on weekends, weekday evenings, and statutory holidays.

A home care nurse may be worried about making a mistake that upsets a parent, but a parent is worried about making a mistake that impacts the safety of their child. We want to know how to solve problems to avoid going to the emergency department, but also how can we stay away from calling [the hospital] again or prevent future ED visits. How can we become more independent.

FC, Patient and Family Advisory Network

Parents would have ongoing support after discharge. Right now, our follow up is limited.

Experienced registered nurse

Having this service would be a huge help to our families overnight and over the weekend.

Experienced registered nurse

Participants also highlighted the need for a service like Connected Care Live to prevent after-hours ED visits and how valuable it is to be able to access video-calling features in addition to text or calling to feel more confident about caring for their child at home.

If I had this service, I’m 99.9% sure, it would have prevented an ED visit.

FC, Patient and Family Advisory Network

Especially for new families, this service would be invaluable. Even just over the phone it can be difficult to walk you through what needs to be done but having the option of the video makes a huge difference to see what you are referring to. To have equipment and supplies available through Connected Care Live and physically see what needs to be done.

FC, Patient and Family Advisory Network

##### Supports Caregiver After-Hours Stress and Strengthens Partnerships

Participants further voiced that having a gap in after-hours services available to FCs has contributed to increased caregiver stress when caring for their child’s medical device at home. By expanding Connected Care Live to families, they described how it would not only help alleviate stress but also enhance existing partnerships with SickKids’ providers.

A lot of the concern I have as a parent to deal with at home is the anxiety of not knowing if you’re doing the right thing or not.

FC, Patient and Family Advisory Network

With each consult encounter, it would strengthen the partnerships we have with our families.

Experienced registered nurse

##### Improves Provider Satisfaction and Reduces Workflow Interruptions

Nurses are often responding to FC inquiries to ensure that they receive the high-quality specialized support that they need to manage their child’s medical devices at home. However, in the absence of formal channels to support families after-hours, nurses voiced the increased workflow interruptions they were experiencing, highlighting the need for a service like Connected Care Live. Particularly, the G-Tube Resource Nurses mentioned that they frequently deal with a large volume of after-hours emails upon returning from the weekend. Because of limited capacity, they are then required to schedule fewer patients and specialized wait-listed procedures on Mondays

Right now, we see many backdoor consults through emails. The increasing number of emails are difficult to organize and manage, but families show a lot of appreciation of this support. This service will provide a more formal structure and secure pathway for us to provide recommendations.

Experienced registered nurse

We often book less patients on Mondays because we are so busy responding to after-hours emails.

Experienced registered nurse

#### ED Visit Data Results

**Figure 3:**
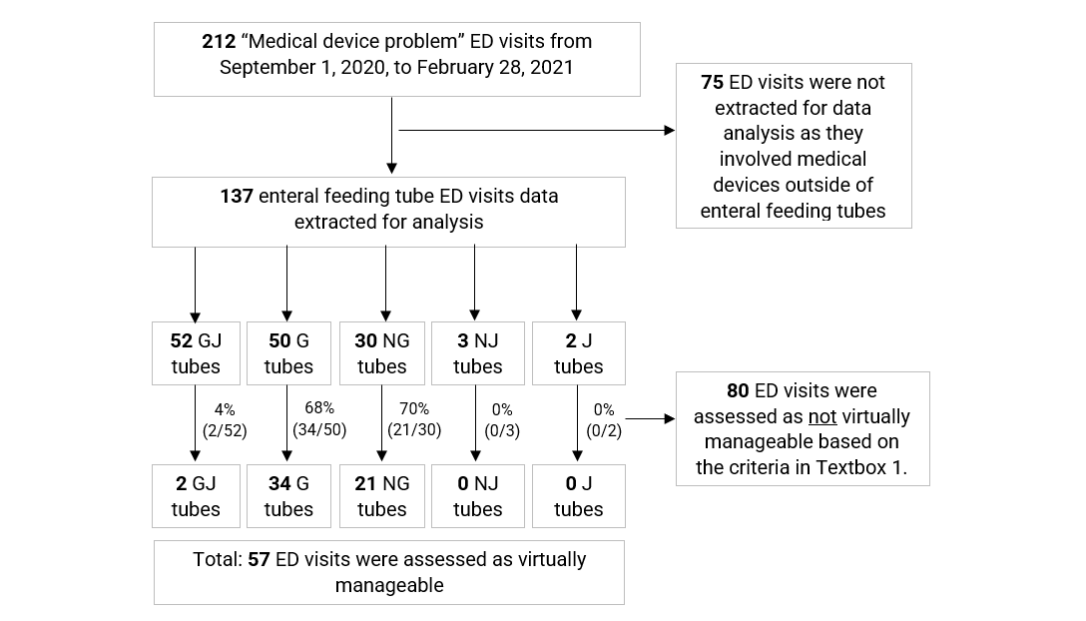
Flow diagram showing EPIC emergency department (ED) visits from September 2020 to February 2021. G tube: gastrostomy tube; GJ tube: gastrojejunostomy tube; NG tube: nasogastric tube; NJ tube: nasojejunal tube; J tube: jejuonostomy tube.

Figure 3 presents a flowchart depicting the findings from the extraction of Epic ED visit data. Of the 212 ED visits categorized as “medical device problems,” enteral feeding tubes accounted for nearly two-thirds (n=137, 64.6%). Across these visits, patients and FCs spent an average of 4.2 hours in the ED. Dislodgment (92/137, 67.2%), blockage (19/137, 13.9%), and stoma issues (8/137, 5.8%) were the top 3 reasons for visits. Among the 137 enteral feeding tube visits, 37.9% (52/137) were related to GJ tubes (with 4%, 2/52, assessed as virtually manageable), 36.5% (50/137) were related to G tubes (with 68%, 34/50, assessed as virtually manageable), 21.9% (30/137) were related to NG tubes (with 70%, 21/30, assessed as virtually manageable), 1.5% (2/137) were related to jejunostomy tubes (with 0%, 0/2, assessed as virtually manageable), and 2.2% (3/137) were related to nasojejunal tubes (with 0%, 0/3, assessed as virtually manageable). Across all the ED visits related to enteral feeding tubes, 41.6% (57/137) were assessed as virtually manageable (see Textbox 1 for exclusion criteria).

#### After-Hours Medical Device Inquiries to G-Tube Resource Team Data Results

Out of 192 enteral tube insertions (ie, G and G-J) performed each year by SickKids’ image-guided therapy (n=170) and general surgery (n=22) teams, 47 after-hours emails and voicemails inquiring about 35 different patients to the G-Tube Resource Team were documented over a 2-week period. The results are presented in Table 3. Approximately three-quarters (33/47, 70%) of inquiries were initiated by FCs, nearly all via email (45/47, 96%), and over two-thirds occurred during the first day of the week (33/47, 70%). Note this “first day” includes 1 Tuesday after a statutory holiday with the highest volume of inquiries addressed after a long weekend (13/47, 28%); 35/47 (75%) inquiries were for G tubes and the rest were for either a GJ tube (9/47, 19%) or not stated (3/47, 6%). The top 3 reasons for contacting the team were appointment related (13/47, 28%), stoma or granulation tissue (11/47, 23%), or dislodgement (3/47, 6%). Over one-half of the issues were considered manageable by the Connected Care team (24/47, 51%; see Textbox 2 for exclusion criteria).

**Table 3:** After-hours medical device inquiries to G-Tube Resource Team results from March 26, 2021, to April 8, 2021, out of 192 new insertions each year.

Variable			Inquiries (N=47), n (%)
**Mode of contact**			
	Email	45 (96)	
	Voicemail	2 (4)	
**Individual/group initiating contact**			
	Family caregiver	33 (70)	
	Point-of-care team	9 (19)	
	Other (group home, image-guided therapy team)	5 (11)	
**Type of medical device**			
	Gastrostomy tube	35 (75)	
	Gastrojejunostomy	9 (19)	
	Not stated	3 (6)	
**Reason for contact**			
	Appointment related	13 (28)	
	Stoma/granulation tissue	11 (23)	
	Dislodgement	3 (6)	
	Other (stoma output, tube size/fit, primary insertion, adaptors and catheters, balloon volume, leakage, old blood from tube, blood work follow-up, re-siting tube, routine follow-up)	20 (43)	

#### Time and Travel Savings Analysis Results

Our study forecasted an annual savings of 478.3 hours for SickKids’ ED, 74.4 hours for G-Tube Resource Nurses, and 890.7 hours and 22,740 km (travel) for families. The estimated total annual time and travel savings are presented in Table 4. To calculate travel savings for families, we estimated the average round-trip distance per hospital visit, the average number of visits per year, and the total number of patients based on the ED visit data.

**Table 4:** The estimated total annual time savings for SickKids Emergency Department, family caregivers, and the Gastrostomy Tube Resource Nurses.

Estimation	SickKids Emergency Department	Family caregivers	Gastrostomy Tube Resource Nurses
The calculation to determine the total number of hours per year (eg, number of visits in a year × hours/visit)	137 enteral medical device EDa visits in 6 months × 2 4.2 hours on average/ED visit	2 hours of estimated total travel time/ED visit 4.2 hours on average/ED visit 3 ED visits/year on average 94 families in 6 months × 2	5 minutes/email 47 emails on average every 2 weeks × 2 × 12 months 1 hour blocked off Monday mornings (or Tuesdays if holidays) from clinic visits to respond to after-hours inquiries × 52 weeks
Total number of hours per year	1150	3497	146
Percentage of virtually manageable visits	× 41.6% (57/137) virtually manageable visits including all enteral feeding tube types (see Figure 3)	× 41.6% (57/137) virtually manageable visits including all enteral feeding tube types (see Figure 3) 564 hours for estimated Connected Care Live consults (3 consults per year/1 hour per consult/94 families in 6 months × 2)	× 51% (24/47) Connected Care Resource Nurse manageable visits
Estimated total annual time savings (hours)	478.3	890.7	74.4

^a^ED: emergency department.

### Operational Feasibility

The estimated program service operational requirements are shown in Table 5. These include approximately 0.1 nursing full-time equivalents for supporting the pilot service expansion and 60 hours for initial-year activities such as professional development training workshops, project management, auditing, quality assurance, and administrative tasks before pilot implementation. Service demand was estimated based on ED visits and after-hours inquiries data. Almost 95.6% (131/137) of consults resulting from enteral feeding tube ED visits and after-hours inquiries occurred within the established Connected Care service hours, which are from Mondays to Thursdays, 7 AM to 11 PM, and Fridays to Sundays, 7 AM to 7 PM.

**Table 5:** Connected Care operational requirements for gastrostomy/gastrojejunostomy tube pilot expansion.

Initial annual operational requirements^a^	Ongoing operational requirements^a^	Annual required FTE^b,c^
274 (137×2) enteral ED^d^ visits/year 41.6% (57/137) virtually manageable (includes all enteral feeding tube types shown in Figure 3) 1 hour on average/consult 53 hours of emails 60 hours (project management, payroll, benefits, professional development training, administrative costs, auditing, and quality assurance)	274 (137× 2) enteral ED visits/year 41.6% (57/137) virtually manageable 1 hour on average/consult 53 hours of emails 40 hours (payroll, benefits, ongoing professional development training, administrative costs, auditing, and quality assurance)	FTE = 37.5 hours/week 1950 hours/year (274×41.6% [57/137] = 114 × 1 hour consult + 53 hours of emails) = 167 Connected Care Resource Nurse hours/year 167/1950=0.085

^a^The total time requirement for initial annual operational and ongoing operational requirements was 228 and 208 hours, respectively.

^b^Approximately 0.1 nursing FTEs are required to support the pilot service expansion.

^c^FTE: full-time equivalent.

^d^ED: emergency department.

### Technical Feasibility

We identified minimal technical requirements in consultation with the Connected Care leadership team by leveraging existing platforms and job aids (eg, practice toolkits and quality assurance procedures). Discussion with privacy, legal, information security, and risk functional department leads identified the following requirements before piloting the service expansion to families: best practices for end user verification methods, password updates, revised terms of use, user agreements, and legal disclaimers before piloting the service.

### Overall Feasibility

Results across the economic, operational, and technical assessments all suggest the pilot service expansion of Connected Care Live to FCs of the enteral feeding tube (ie, G, GJ, NG tubes) population to be a feasible virtual care solution to improve FC experiences, support safer hospital-to-home transitions, and reduce after-hours ED visits. All 3 data sources, ED visits, after-hours inquiries, and semistructured interviews, highlighted the gap in after-hours services available to FCs of CMC-TD after discharge to support medical device prevention and management of complications. However, to successfully launch this pilot with the enteral feeding tube population, we acknowledge the need to ensure that robust practice development and privacy, security, and legal best practices are implemented in advance. Service volumes and timing must also be measured throughout to ensure the current on-call operational model is sustainable and cost-effective for future program operations.

## Discussion

### Principal Findings

This study suggests that Connected Care Live is feasible to pilot with FCs of children with newly inserted enteral feeding tubes (ie, G, GJ, and NG tubes), given the significant time and travel savings forecasted, widespread benefits identified during interviews, and minor operational and technical program changes required to support this population. Although limited in scale, this study addresses an important gap in the delivery of nurse-led virtual care services that are specifically offered after-hours and to FCs of the enteral feeding tube pediatric population. Other studies have highlighted the value of nurse-led virtual care services and increasing after-hours access to adult patients to reduce low-acuity ED visits, but only a few studies have investigated after-hours support available to FCs of the pediatric complex care population, a group known for being the highest users of pediatric health care resources [21,22].

During our semistructured interviews, FCs described the increased stress they experienced after-hours beyond discharge, the guilt they felt for interrupting hospital operations when calling in-patient clinical staff, and their dependence on visiting the ED to ensure the safety of their children. Similar findings have been noted in other studies exploring the health and well-being of caregivers for CMC-TD, demonstrating a high level of mental health burden among this population, particularly in the first few weeks following medical device insertions [23,24]. Our interview results also suggest that having 24/7 real-time virtual access to trusted providers to support troubleshooting medical device complications would be valuable for improving hospital-to-home transition experiences, reducing FC after-hours stress, and preventing ED visits. This finding was consistent with other studies, where having 24/7 real-time virtual support and access to trusted providers led to reduced ED utilization rates and high levels of caregiver satisfaction [2,25]. FCs expressed that one of the appealing aspects of real-time virtual support through Connected Care Live was its video-calling feature, which provided visual cues for medical device management. This was seen as an advantage over existing email and phone services. Similarly, an Ontarian telehome care study indicated that parents of CMC-TD had a strong preference for and satisfaction with videoconferencing provider visits to support hospital-to-home transitions [26].

Interview results further highlighted the significant workflow interruptions nurses experienced for nonurgent cases, by either responding to FC after-hours inquiries or ED visits. During an interview with clinicians in the G-Tube Resource Team, they mentioned that they routinely set aside the first hour of every Monday morning, dedicating it to addressing medical device inquiries from families instead of seeing patients in the clinic. This finding suggests that introducing Connected Care Live offers the potential to offset workload and increase capacity to support additional clinic visits during this time. Our interviews with ED nurses demonstrated how frequently parents would arrive at the ED with their children for minor and non–life-threatening enteral feeding tube complications. These visits often required consultation from Connected Care and the G-Tube Resource Team or minimal intervention, which in both cases constrained resources and delayed care of other higher-acuity cases in the ED. This finding was consistent with other studies in which increased waiting times, overcrowding, and reduced quality of care were seen across EDs with a high census of low-acuity patients [27].

In our ED visits analysis, high economic feasibility was established among the enteral feeding tube population, as they accounted for the largest proportion of patients presenting with medical device complications (137/212, 64.6%), with 41.6% (57/137) of these visits assessed as virtually manageable or considered “avoidable.” Of the 137 enteral feeding tube visits, the largest proportion of visits was found among patients with G tubes (50/137, 36.5%) and GJ tubes (52/137, 37.9%), whereas the highest percentages of visits assessed as virtually manageable were found with G tubes (34/50, 68%) and NG tubes (21/30, 70%). These findings are consistent with G tube insertions being one of the most common pediatric surgeries [28] and enteral feeding as one of the most commonly performed procedures by FCs of CMC-TD at home [7]. Given this large volume of potentially virtually manageable visits, we anticipate that more than half of enteral feeding tube complications would not require ED provider intervention and could be successfully troubleshooted at home with video-enabled access to expert support that builds confidence and competence for FCs [4]. This finding was comparable with other studies, which highlight that pediatric patients with newly inserted G tubes typically present to the ED with low-acuity complications that do not require admission and can often be supported in home and community care settings by specialized family education and reassurance [4,5,29].

In this study, the question of which types of ED visits were potentially avoidable and otherwise virtually manageable was explored to prepare an iterative plan for safe and impactful service expansion. G tube complications were identified as highly preventable and virtually manageable at home based on well-established practice standards amenable to virtual care. GJ tubes had a low percentage of visits assessed as suitable for virtual management; however, they accounted for the highest proportion of ED visits related to enteral feeding tube complications. Previous studies have demonstrated similar findings in which GJ tubes accounted for some of the highest proportions of device-related ED encounters requiring hospitalization, with reductions in complications over time with additional supports (eg, complex care program enrollment) [4,30]. NG tubes were identified as highly preventable; however, existing literature demonstrates an increased risk for complications and a lack of confidence in best practices associated with managing complications in home and community care settings compared with G tubes [31]. This finding suggests the need for after-hours support to reduce preventable visits. In addition, with recent professional practice guidelines limiting the requirement for imaging of NG tubes, suitability for virtual care services is anticipated [32].

The operational requirements identified by study participants indicated that minimal program changes would be required to support the service expansion. Based on the consultation volume and timing estimates, the Connected Care program could support the pilot study with existing clinical staff, who would already be familiar with navigating this online platform. However, future specialty training for Connected Care Resource nurses would be required to reinforce education on the management of enteral feeding tubes, while mitigating risks to nursing practice with gaps in knowledge as identified during interviews. Having ongoing professional development training opportunities for nurses is widely recognized to improve the quality of care and patient safety needed to promote best practices among FCs [33]. As highlighted above, across the different enteral feeding tube types, there is the highest feasibility to pilot the service initially with G and GJ tube patients, given the existing support available from the G-Tube Resource Team and the quality of evidence–based resources. This team plays a critical role in the success of this pilot through their support for building content for training workshops in collaboration with Connected Care Resource Nurses, and for providing expertise as needed throughout the pilot Go-Live period.

While many CMC-TD receive home and community care services from regulated care providers to support medical device management, these services are often not delivered around the clock. To ensure the continuing safety and quality of care provided at home, unregulated care providers including FCs play an important role in filling this service gap and building competencies in caring for their child’s home care technologies [34]. In acknowledging these legal implications, during our technical feasibility assessment and semistructured interviews discussing the risks of the proposed service, we identified the need to update language around accountability for care in the Connected Care Live Terms of Use to better reflect both regulated (home and community care providers) and unregulated care providers (FCs). For example, when outlining the purpose of the service intended to aid and support users, both health care providers and FCs were separately addressed to highlight that information shared through the service is not a substitute for neither professional judgment nor services delivered by health professionals within a patient’s circle of care [35].

Overall, the study findings demonstrated a significant gap in after-hours resources available to FCs of CMC-TD beyond discharge. This real-time nurse-led virtual care service offers a valuable approach to improving FC after-hours experiences and reducing avoidable health care encounters. In the future, upon successful pilot study and evaluation of Connected Care Live with the G and GJ tube population followed by that with the NG tube population, this service holds the potential to be scaled to a much larger group of medical devices and families spanning beyond the Greater Toronto Area.

### Strengths and Limitations

The greatest strength of this study was the widespread commitment and interest on behalf of FCs, nurses, and hospital leadership to establish the feasibility of piloting the Connected Care Live service expansion. Although a small sample of FCs and nurses were interviewed, the results signaled the need for additional after-hours support. Some of the study limitations were that time and travel savings data may have been impacted by the COVID-19 pandemic and seasonality with variable ED visit volumes; however, other factors were considered, such as pandemic-related decreases in home care visits further straining FC support at home. In addition, manual data extraction and collection of ED visits and after-hours inquiries data were required to ensure timely access to data. For the after-hours medical devices inquiries, we would also like to acknowledge that the G-Tube Resource Team primarily supports the G and GJ tube population, thus excluding the NG tube population from this portion of this analysis.

In addition, semistructured interviews were focused internally across SickKids stakeholders, and future studies would largely benefit from including external perspectives, such as home and community care providers. While the primary focus for service expansion in this study was FCs, future studies would also benefit from interviewing patients with new medical technologies themselves to better understand the direct impact this type of service has on their hospital-to-home transition and after-hours experiences. Some of the unaccounted time savings for the economic feasibility analysis included the amount of time spent by Connected Care leadership on the feasibility study, pilot project planning and evaluation, and redundant resourcing for safety and practice development during the initial pilot Go-Live period. Other considerations for the time savings calculations are identifying the number of nurses required in the G-Tube Resource Clinic during their first hour of work and the number of patients that can be seen in an hour.

### Conclusions

The findings from this study were successful in demonstrating the economic, operational, and technical feasibility before launching the pilot expansion of Connected Care Live initially to FCs of children with newly inserted G and GJ tubes, followed by NG tubes. This nurse-led virtual care consultation service for families serves as a promising tool to promote safe and positive hospital-to-home transition experiences, while reducing after-hours ED visits. In future studies, further investigation will be needed to establish the feasibility of scaling such a service to other medical devices (eg, central lines, tracheostomies) and geographic areas across Ontario.

## Abbreviations

CMC-TD: children with medical complexity and technology dependence
ED: emergency department
FC: family caregiver
FTE: full-time equivalent
GJ tube: gastrojejunostomy tube
G tube: gastrostomy tube
NG tube: nasogastric tube
NJ tube: nasojejunal tube
J tube: jejuonostomy tube
